# The Crystallography of Enzymes: A Retrospective and Beyond

**DOI:** 10.3390/cryst15110966

**Published:** 2025-11-08

**Authors:** Tianyi Huang, Jannat Khan, Sheryar Lakhani, Albert Li, Aditya Vyas, Julia Hunt, Sara Andrea Espinosa Garcia, Bo Liang

**Affiliations:** Department of Biochemistry, Emory University School of Medicine, Atlanta, GA 30322, USA

**Keywords:** crystallography, enzymes, enzyme commission (EC) classes, structural analysis, protein data bank (PDB), enzymology, retrospective

## Abstract

Crystallography plays a crucial role in understanding the functions of macromolecules by determining their three-dimensional structures at the atomic level. This review outlines the history of crystallization, explains the principles of crystallization, and provides a comprehensive retrospective on the role of crystallography in enzymology, with a particular focus on the seven Enzyme Commission (EC) classes. For each class, we highlight representative enzymes and the specific mechanistic insights enabled by crystal structures, oxidoreductases (the “yellow enzyme” lineage), transferases (phosphotransferase systems), hydrolases (RNase III and chymotrypsin), lyases (fumarase), isomerases (pseudouridine synthases), ligases (E3 ubiquitin ligases), and translocases (ATP synthase), emphasizing cofactor usage, conformational change, regulation, and implications for disease and drug discovery. We also compile EC-wide statistics from the Protein Data Bank (PDB) to quantify structural coverage. The limitations and challenges of current crystallization techniques are addressed, along with alternative experimental methods for structural elucidation. In addition, emerging computational tools and biomolecular design are also discussed. By reviewing the trajectory of enzymology and crystallography, we demonstrated their profound impact on biochemistry and therapeutic discovery.

## 1. Introduction

Crystals are solid materials whose atoms or molecules are arranged in a highly ordered, repeating pattern extending in all three spatial dimensions. This arrangement makes crystals ideal for structural studies. The process by which crystals form, known as crystallization, plays a significant role in modern-day biological sciences for the structural analysis of various biomolecules [1].

### 1.1. Historical Development of Crystallography

**Figure 1.**
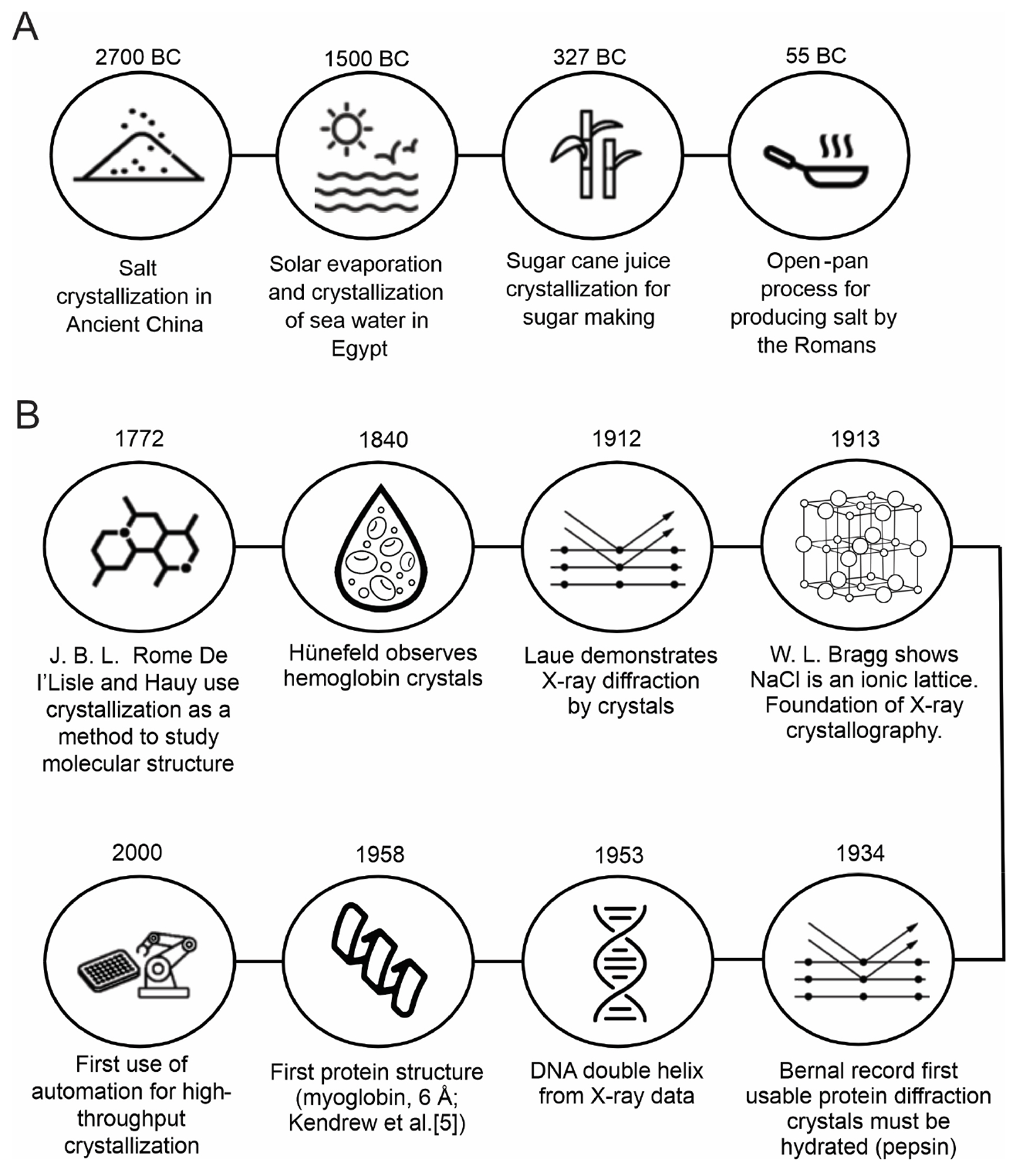
Crystallization History [2,5]. (**A**) Ancient history of crystallization. Illustrates the foundations of crystallography over 2700-55 BC. (**B**) Modern-day evolution of crystallization techniques, including early protein isolation and optimization.

The practice of crystallization has deep historical roots. Procedural details of crystallization used to make salt were documented in ancient Chinese writings dating back to 2700 BC, appeared in Egyptian records around 1500 BC, and were recorded in Roman records as early as 55 BC. Ancient Egyptian records also show the crystallization of sugar cane juice to create preservatives (Figure 1A). These practices with purely practical purposes laid the groundwork for scientific exploration, which began around the start of the 19th century, with pioneers like Romé de L’Isle and René Just Haüy applying crystallization to study the geometric properties of minerals. These early studies helped define crystallization as a replicable physical process, paving the way for its application in molecular biology [2] (Figure 1B).

One of the most significant scientific breakthroughs came with the development of X-ray crystallography. Crystallography is the quantitative determination of atomic structure from diffraction by a crystal’s repeating lattice. First performed by Max von Laue et al. in 1912, the method relies on the diffraction of X-rays by a well-ordered crystal lattice, yielding electron density maps that can be interpreted to build detailed molecular models. William Lawrence Bragg, who won the Nobel Prize in Physics in 1915, is most famous for his Bragg’s law on the diffraction of X-rays by crystals. In 1913, it was first discovered that NaCl crystals are not composed of molecules, but rather patterns of ions. The famous discovery served as the foundation of X-ray crystallography [3].

### 1.2. Principles of Crystallization

While the technique was developed to probe the structure of ionic lattices, crystallization was also quickly applied to organic and biomolecules at the same time. Notably, in 1840, Friedrich Hünefeld observed the crystallization of hemoglobin from earthworm blood, marking the first recorded instance of a protein crystallizing [4]. By the late 19th and early 20th centuries, many biomolecules, including proteins, RNA, and DNA, had been successfully crystallized.

Determining the structures of these macromolecular crystals, however, required further breakthroughs. In 1934, J.D. Bernal discovered that protein crystals must be kept hydrated to yield useful diffraction patterns, laying the groundwork for the development of protein X-ray crystallography. Several years later, the phase problem for proteins was gradually overcome, and in 1958, John Kendrew and colleagues reported the first-ever protein structure of myoglobin at 6 Å resolution. As a result of these breakthroughs, he received the 1962 Nobel Prize in Chemistry for his studies on the structures of globular proteins [5]. In addition to proteins, another landmark was the discovery of the DNA double helix in 1953 by Watson and Crick, based on X-ray diffraction data [6].

**Figure 2.**
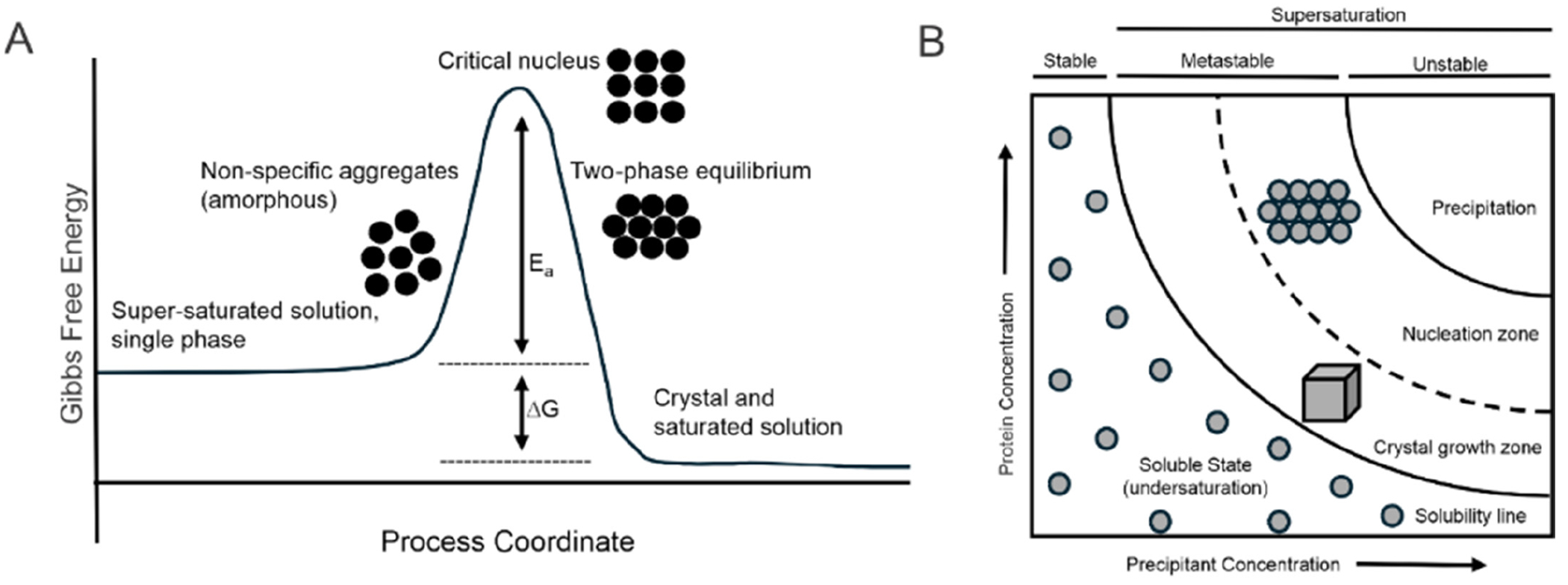
Schematic illustrations of a Free Energy and Phase Diagram. (**A**) Free Energy Diagram illustration. The formation of a critical nucleus requires overcoming an energy barrier. After the nucleus forms, crystal formation becomes more thermodynamically favored, allowing the crystals to grow spontaneously. (**B**) Crystallization phase diagram. Ideal crystal formation occurs in the metastable zone of crystal growth. Parameters such as protein and precipitant concentration, pH, and temperature can be adjusted to change the phase of the protein solution into the desired phase.

While widely used in structural biology today, protein crystallization remains a delicate process. Crystallization begins with the formation of a critical nucleus and the achievement of supersaturation, as illustrated by a free energy and phase diagram (Figure 2). There are four zones in the phase diagram: a supersaturation zone where the protein will precipitate, a moderate supersaturation zone where nucleation will occur, a lower supersaturation zone where crystal growth can occur, and an undersaturated area where the protein is stable and will never crystallize. The supersaturation state is achieved by adding precipitating agents, such as neutral salts and polymers, to the solute and solvent [7]. When the concentration of the protein and the precipitant exceed the solubility limit, supersaturation is achieved, and crystallization may occur if all other conditions are favorable for nucleation and crystal growth [7]. Ideal crystal formation occurs in the metastable zone where no further nucleation will take place, but large, organized crystals can form. The formation of the critical nucleus in the metastable zone requires overcoming a significant energy barrier. As non-specific aggregates grow, protein molecules continue to bind, leading to the formation of the critical nucleus, which can either continue to grow or fall apart. The crystal growth after critical nucleus formation is more thermodynamically favored [8] (Figure 2A). In reaching this metastable state, phase separation occurs, where proteins transition from their soluble state to a solid state [9]. In the solid state, protein molecules form critical nuclei, and the protein concentration decreases, initiating a growth phase [9]. Protein molecules then continue to bind to the nuclei to form a crystal until the protein concentration in the solution dissipates [9] (Figure 2B).

### 1.3. Practical Crystallization Methods

**Figure 3.**
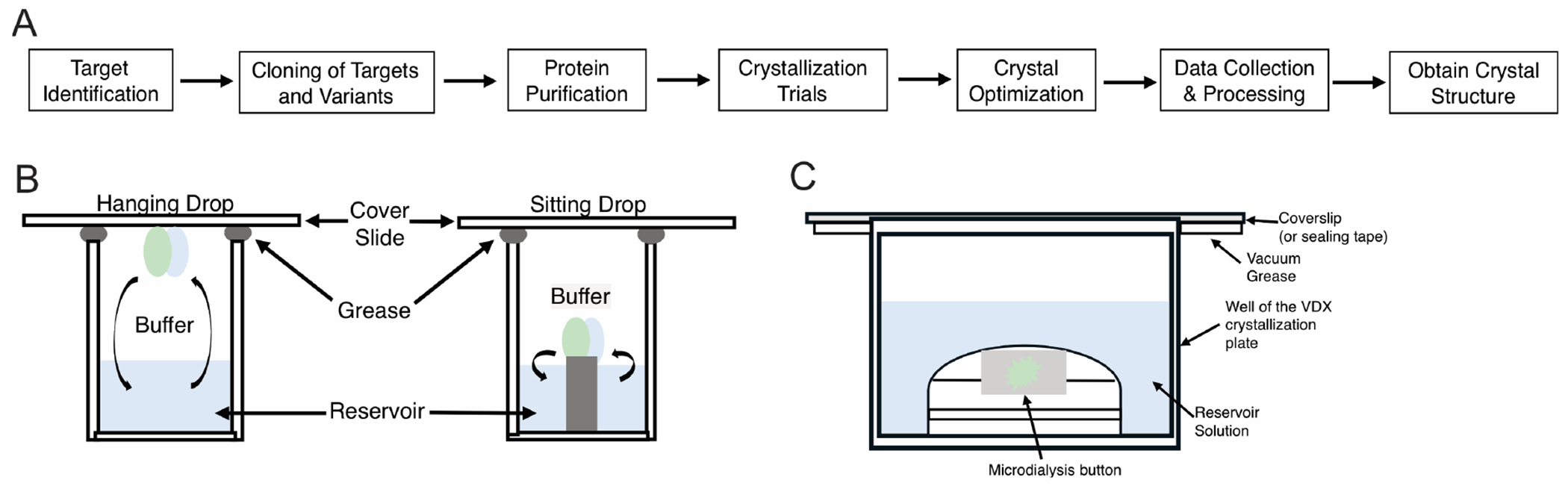
Crystallization Methods. (**A**) Crystallization flowchart showing the steps from target identification to obtaining a crystal structure. The protein purification and crystallization optimization steps are the most time-consuming and complex steps. (**B**) Hanging drop (**left**) and sitting drop (**right**) are both vapor diffusion methods. Both methods utilize buffer exchange between the protein drop and the reservoir. (**C**) The microdialysis method utilizes a semi-permeable membrane that slowly mixes the precipitant solution with the protein sample.

Key methods for inducing crystallization include vapor diffusion and dialysis, both of which offer controlled environments to promote crystal formation (Figure 3). Vapor diffusion requires a constant exchange between the protein solution, often represented as a hanging or sitting drop, and a reservoir solution containing crystallization agents [10]. The reservoir solution is then set to a higher or lower concentration than the protein solution to push the system towards equilibrium [10]. Dialysis, by contrast, separates proteins from precipitants using semi-permeable membranes to enable slower equilibration [11].

**Figure 4.**
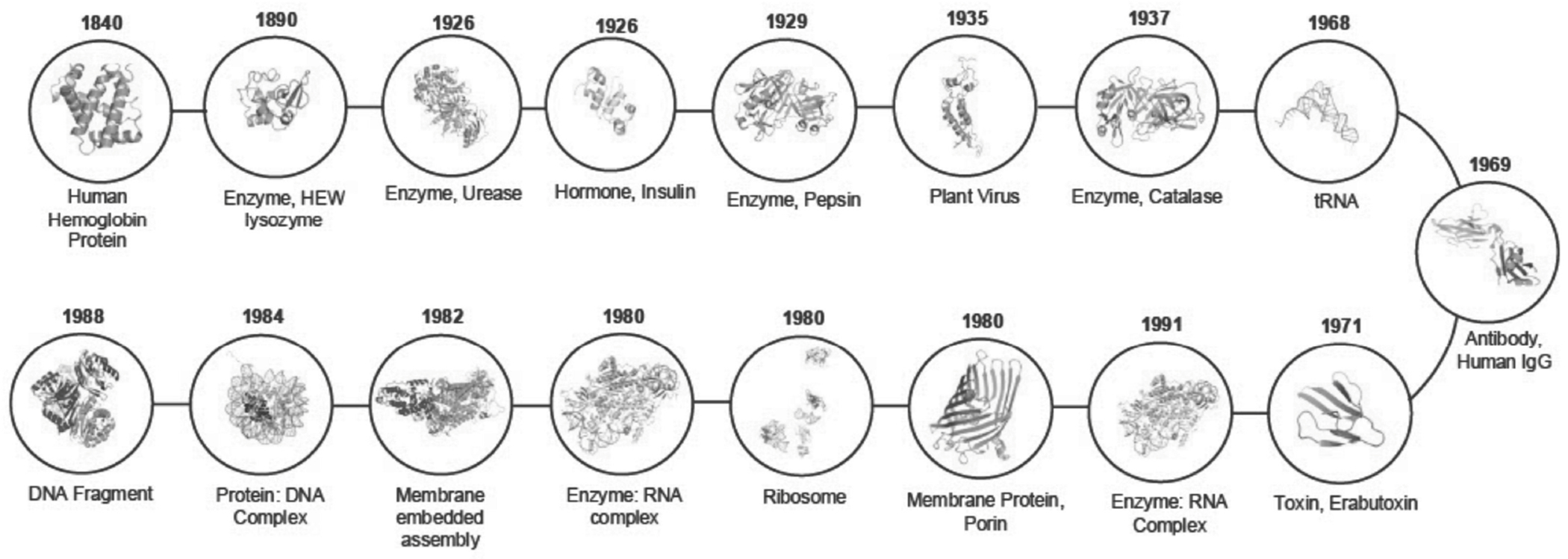
Timeline of obtaining 3D structures for various crystallized macromolecules. 1840, hemoglobin protein; 1890, enzyme hen egg-white (HEW) lysozyme; 1926, enzyme urease; 1926, hormone insulin; 1929, enzyme pepsin; 1935, plant virus; 1937, enzyme catalase; 1968, tRNA; 1969, antibody human IgG; 1971, toxin erabutoxin; 1980, enzyme–RNA complex; 1980, ribosome; 1980, membrane protein porin; 1982, membrane-embedded assembly; 1984, protein–DNA complex; 1988, DNA fragment; 1991, Enzyme: RNA Complex. All molecular graphics were generated using PyMOL 3.1.6 Molecular Graphics System, Schrödinger, LLC (New York, NY, USA).

As the late 20th century progressed, crystallization was increasingly applied to more macromolecule classes, including proteins, nucleic acids, viruses, and even ribosomes, among which the most biochemically and medically significant targets of crystallographic investigation are enzymes (Figure 4). Enzymes are essential for many biological reactions, acting as biological catalysts. While most enzymes are proteins, some RNA-based enzymes, known as ribozymes, also exist, but they function in a slightly different manner [12].

### 1.4. Insights into Enzymology

Enzyme specificity makes them invaluable in research, diagnostics, and therapeutics. Early models include the “lock and key” model suggested by Emil Fischer, which was later refined and replaced by the “induced fit” model, which better explains how enzymes accommodate substrate groups, bond types, and stereochemistry through conformational changes upon binding [13]. Furthermore, enzymes often require cofactors (inorganic ions) or coenzymes (organic molecules) to facilitate the catalysis of reactions. They may also have regulatory sites that bind small molecules, substrates, or intermediates, modulating activity through feedback mechanisms. Enzyme inhibitors can reduce or block catalysis by binding reversibly or irreversibly to active or allosteric sites.

Enzymes generally participate in acid-base and metal-ion catalysis, and other mechanisms yielding proximity/orientation effects. The seven classes of enzymes include hydrolases, oxidoreductases, transferases, lyases, isomerases, and ligases. Following the boom in enzyme discovery, an ad hoc committee under the International Union of Biochemistry established a classification system in the 1950s. This led to the establishment of a numbering system comprising the enzyme class, subclass, and sub-subclass [14]. Enzymes are categorized into seven different enzyme classes, known as Enzyme Commission (EC) classes, with subclasses and sub-subclasses in each. They are classified into seven classes based on enzyme function and the type of reaction they catalyze. Each enzyme is given four numbers. The first number identifies the EC class, and the following numbers provide more specificity to the enzymes’ function [15].

**Figure A1.**
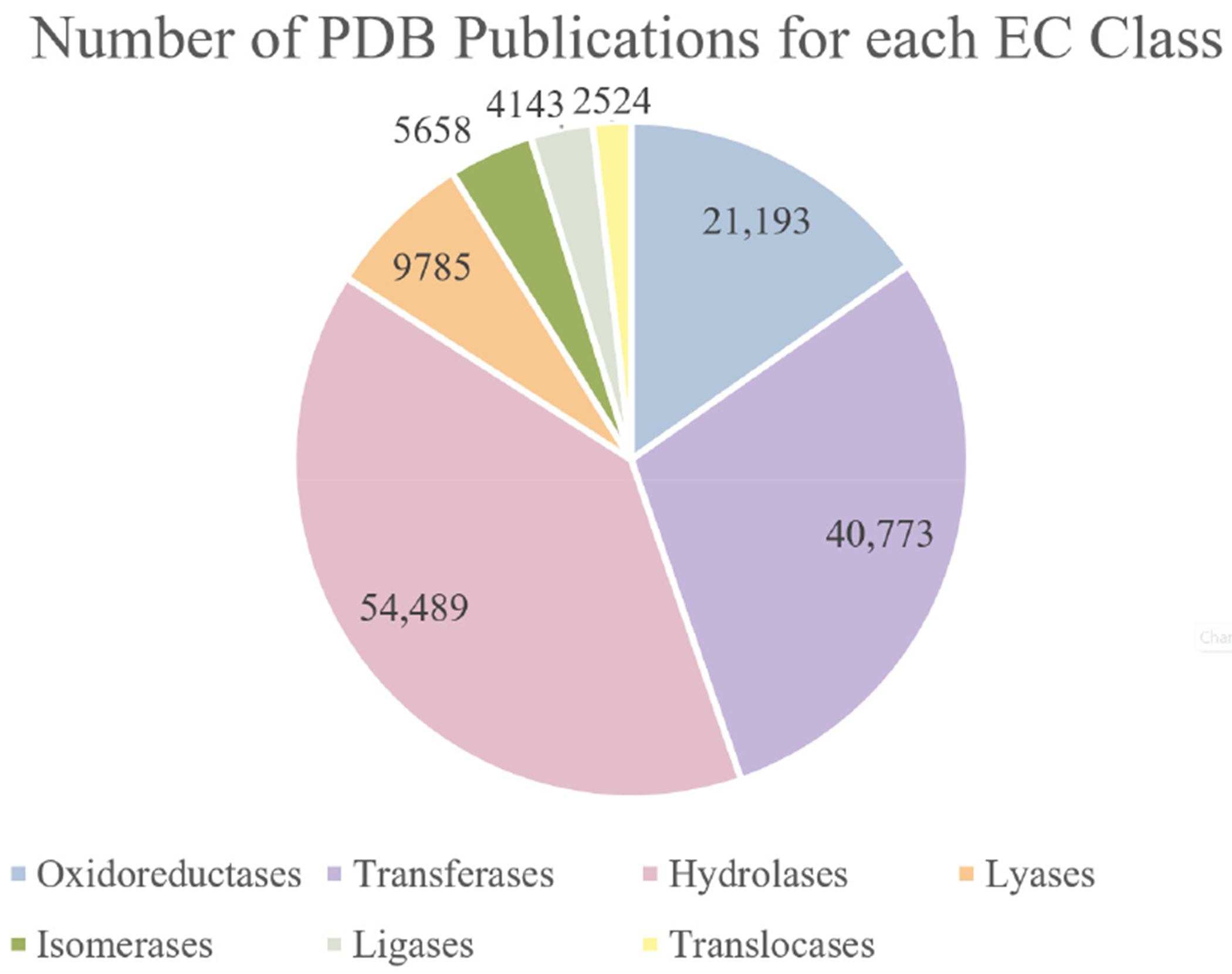
Number of PDB publications for each enzyme class (updated by 31 July 2025). Oxidoreductases (EC 1): 21,193 publications; Transferases (EC 2): 40,773; Hydrolases (EC 3): 54,489; Lyases (EC 4): 9785; Isomerases (EC 5): 5658; Ligases (EC 6): 4143; Translocases (EC 7): 2524. Data was obtained from the Research Collaboratory for Structural Bioinformatics (RCSB) Protein Data Bank (PDB).

This review provides insights into each of the seven EC classes, highlighting the major discoveries associated with each. In the following sections, we review the seven Enzyme Commission (EC) classes (EC 1–7), oxidoreductases, transferases, hydrolases, lyases, isomerases, ligases, and translocases, with brief overviews and representative crystallographic case studies. To provide a structural overview of this classification, we compiled statistics from the Protein Data Bank (Figure A1). As summarized in the tables, hydrolases and transferases dominate in terms of structural coverage, whereas ligases and translocases remain underrepresented, highlighting areas where structural biology still faces challenges (Supplementary Tables S1–S8).

## 2. Crystallographic Insights into Enzyme Mechanisms

### 2.1. Oxidoreductases

Oxidoreductases are a class of enzymes that transfer electrons from one molecule to another, assisting oxidation-reduction reactions. They are categorized as EC 1 in the enzyme classification system. They are especially significant for biochemical reactions, including glycolysis, where an oxidoreductase named glyceraldehyde-3-phosphate dehydrogenase (EC 1.2.1.12) reduces nicotinamide adenine dinucleotide (NAD+) to nicotinamide adenine dinucleotide with hydrogen (NADH), directly facilitating the crucial process of energy production [16].

Underscoring the invaluable contributions of oxidoreductases in the body, the 1955 Nobel prize for physiology or medicine was awarded to Hugo Theorell for his work on a riboflavin enzyme then referred to as the ‘yellow enzyme’. This journey began in the 1930s due to the growing interest in the role of oxidoreductases in energy and metabolism, and the yellow ferment enzyme was later confirmed to be NADPH dehydrogenase. The enzyme presented itself as a great, research-viable candidate due to the fading of color upon reduction and its resurfacing upon oxidation [17].

Theorell had joined forces with Otto Warburg further to explore the characteristics of the yellow ferment enzyme. At the Warburg lab, he developed an electrophoresis machine to separate different parts of the enzyme. Through this technique, he determined that the molecular weight (M.W.) of the proteinogenic enzyme was 75,000 Dalton and delineated its protein and prosthetic group components, namely the Vitamin B2 coenzyme [18]. These results highlighted the crucial fact that components were inactive on their own but displayed catalytic activity when combined.

**Figure 5.**
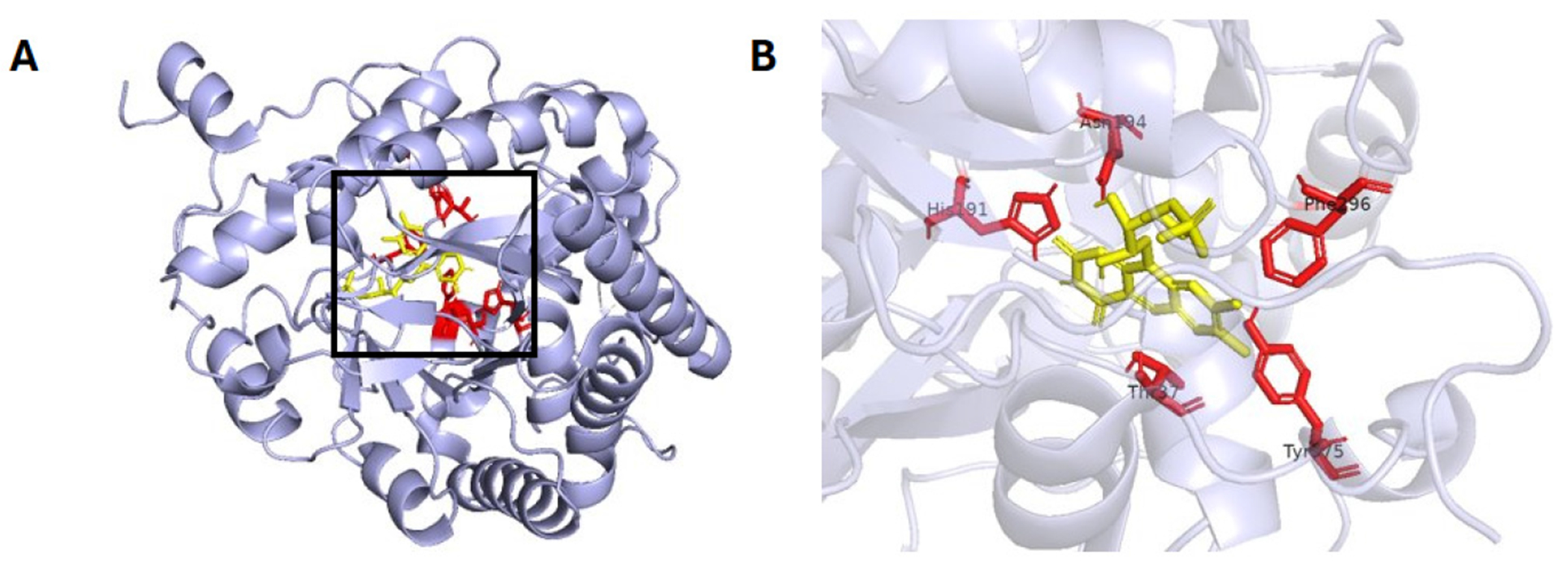
PDB structures of 1OYC (Old Yellow Enzyme) monomer. (**A**) The protein monomer is shown in light blue, with the flavin mononucleotide (FMN) cofactor in yellow at the core of the fold. Key active-site residues are shown in red (Thr37, His191, Asn194, Phe296, and Tyr375). (**B**) A zoomed in view of the active site (the region included in the black frame in panel (**A**)). His191 and Asn194 form a hydrogen-bonding pair that puts nicotinamides, phenols and steroids on the si-face of the flavin. Thr37 donates a backbone hydrogen bond to N5 of FMN. Phe296 and Tyr375 surround dimethylbenzene edge of the flavin. All molecular graphics were generated using PyMOL 3.1.6.

X-ray crystallography was crucial for revealing the structural basis of its “yellow” flavin chemistry. Optimized crystallization of yeast old yellow enzyme (OYE) yielded large, well-diffracting crystals [19], which enabled 2.0 Å structures of the oxidized and reduced enzyme (PDB ID: 1OYC) (Figure 5) [20]. Subsequent high-resolution OYE-family structures generalized these features and revealed alternative arrangements that tune active-site electrostatics and substrate capture [21].

### 2.2. Transferases

Transferases are a class of enzymes that move functional groups from a donor to an acceptor, carrying out group-transfer reactions. They are categorized as EC 2 in the enzyme classification system. By transferring chemical marks on proteins, nucleic acids, and lipids, transferases underpin cellular information processing, signal transduction, and epigenetic regulation [22].

**Figure 6.**
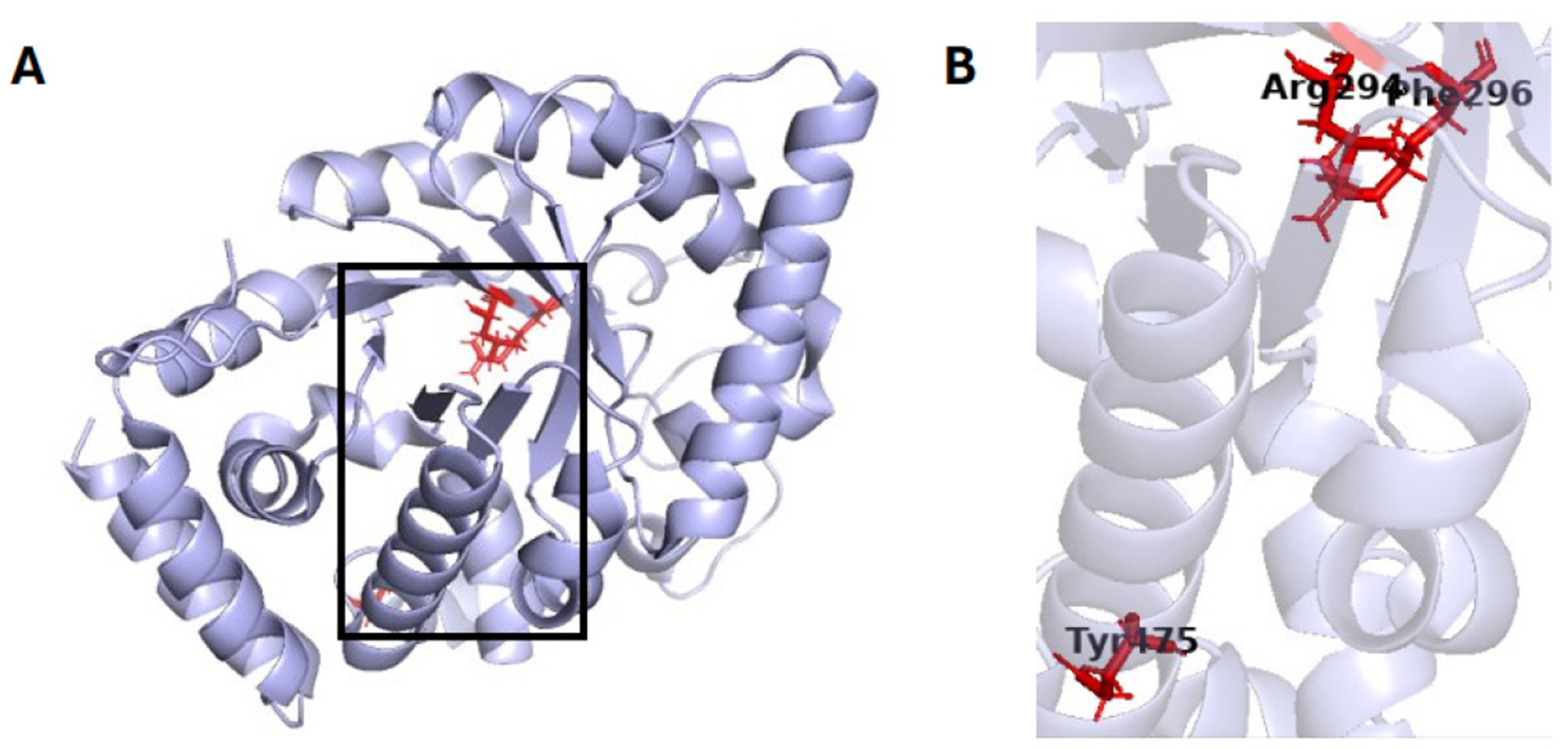
PDB structures of 6VBJ (Enzyme I, C-terminal EIC domain). (**A**) The protein monomer is shown in light blue. Key active-site residues are shown in red (Arg294, Phe296, and Tyr475). (**B**) A zoomed-in view of the active site (the region included in the black frame in panel (**A**)). Arg294, Phe296, and Tyr475 form the pocket that positions the phosphoenolpyruvate (PEP) substrate for nucleophilic attack. All molecular graphics were generated using PyMOL 3.1.6.

A classic example of transferases is the bacterial phosphoenolpyruvate (PEP) in carbohydrate phosphotransferase system (PTS) (EC 2.7.1), which couples glucose uptake to its phosphorylation in a cascade involving Enzyme I (EI) (PDB ID: 6VBJ) (Figure 6), the histidine phosphocarrier protein (HPr) (PDB ID: 1PFH), and sugar-specific Enzyme II (EII) (PDB ID: 1F3G) complexes. EI autophosphorylates on a conserved histidine using PEP, then transfers the phosphoryl group to HPr, which in turn donates it to the EIIA domain of the membrane-spanning EII complexes [23].

High-resolution crystal structures of EI from *E. coli* revealed a symmetric dimer with two magnesium-dependent active sites that undergo large domain movements upon phosphorylation, highlighting how conformational changes drive phosphoryl transfer between domains. Likewise, the *E. coli* EIIA component was shown to adopt a Rossmann-like fold that docks onto HPr via a conserved interface, explaining the specificity and regulation of sugar uptake. Most recently, the structure of the EIIC glucose transporter from *E. coli* demonstrated an inward-open conformation, with key sugar-binding residues lining a transmembrane cavity, providing a molecular basis for substrate recognition and gating during transport [23].

Together, these crystallographic insights into the multi-enzyme PTS illustrate how sequential phosphoryl transfer and conformational changes coordinate group translocation across the membrane, emphasizing both metabolic regulation and cellular adaptation to nutrient availability.

### 2.3. Hydrolases

Hydrolases are a class of enzymes that cleave chemical bonds by the addition of water, catalyzing hydrolysis reactions [24]. They are categorized as EC 3 in the enzyme classification system. By converting substrates into lower-energy products and releasing functional groups, hydrolases enforce pathway irreversibility and shape metabolic flux, playing prominent roles in proteolysis, lipid turnover, carbohydrate degradation, and phosphoester/phosphoanhydride hydrolysis [25].

Ribonuclease 3 (RNase III) (EC 3.1.26.3) plays a critical role in overall rRNA and mRNA processing, as it is responsible for cleaving double-stranded RNA molecules [26]. RNase III belongs to a family of endoribonucleases and is known to be involved in the maturation of both prokaryotic and eukaryotic RNAs. RNase III and other RNase enzymes play a crucial role in activating RNA molecules to initiate various activities, such as protein synthesis and gene regulation [26]. RNase III binds to the 3′ end of RNA and can facilitate a single nick or a double-strand break, depending on the degree of base pairing present in the substrate. Being classified as a 3.1 enzyme class, RNase III cleaves ester bonds and activates water as a nucleophile to hydrolyze its target to create both 3′ and 5′ ends of a cleaved RNA strand [27].

**Figure 7.**
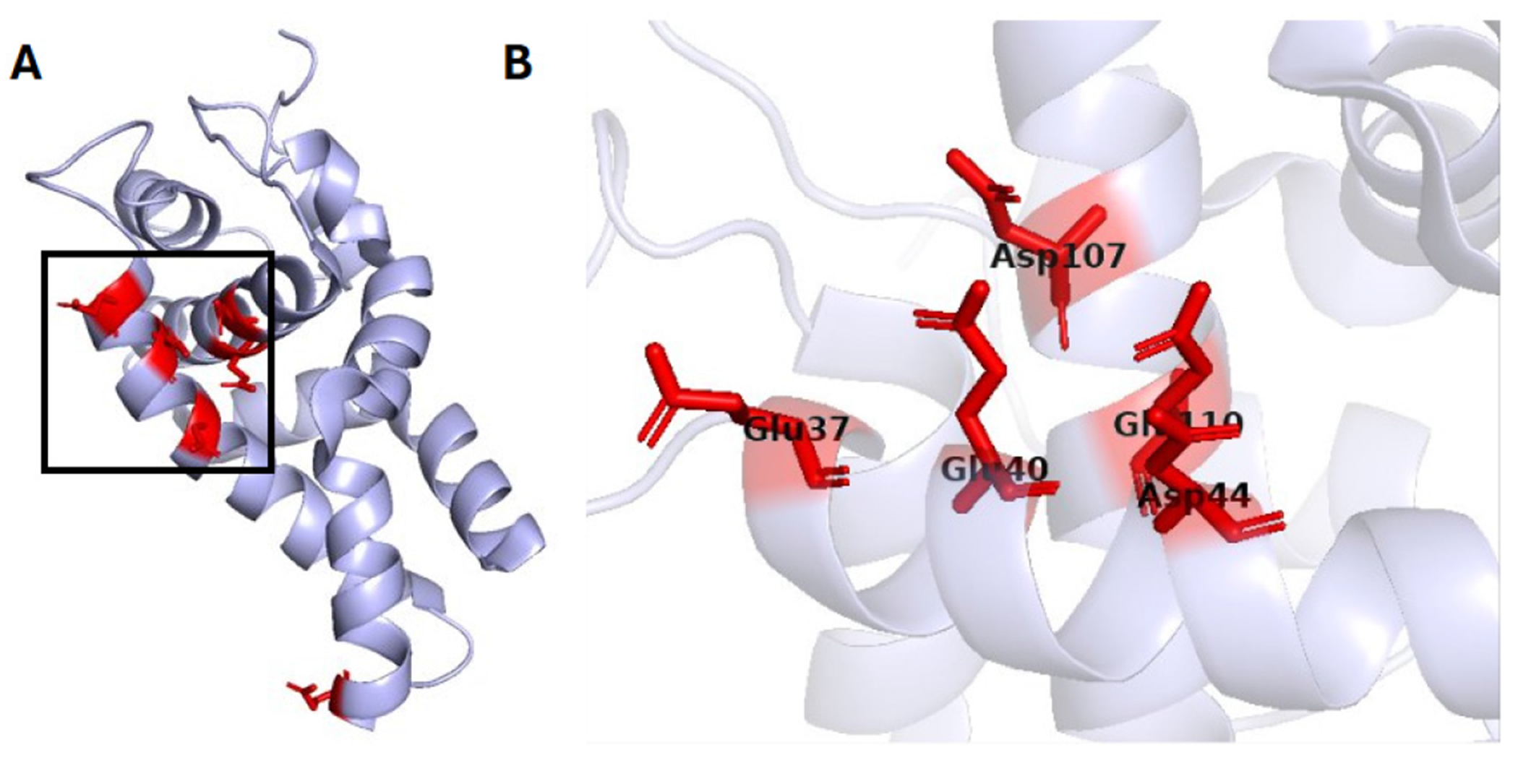
PDB structures of 1I4S (RNase III endonuclease domain). (**A**) The protein monomer is shown in light blue. Key active-site residues are shown in red (Glu37, Glu40, Asp44, Asp107, and Glu110). (**B**) A zoomed-in view of the active site (the region included in the black frame in panel (**A**)) reveals the clustered acidic residues that form the compound catalytic center responsible for double-stranded RNA cleavage. These residues coordinate metal ions (Mg^2+^ or Mn^2+^) and activate water molecules for nucleophilic attack on the phosphodiester bond, forming characteristic RNA fragments with 3′ overhangs. All molecular graphics were generated using PyMOL 3.1.6.

The catalytic activity of RNase III is supported by the RNase III domain, which has also been crystallized and is known to be approximately 150 amino acids in length, comprising around seven α-helices. The first reported RNase III domain structure was obtained through crystallization of Aquifex aeolicus (PDB ID: 1I4S) (Figure 7), which revealed α-helices, a dimeric structure, and divalent metal ions in each subunit [28,29]. Further structural details were obtained from the crystallization of RNase III via Mycobacterium tuberculosis, providing a deeper analysis of the RNase III domain structure. RNase III has been crystallized in multiple organisms, ranging from bacteria to mammals, as its critical role in maturing RNA is essential to all organisms [30].

Another important enzyme in this class is Chymotrypsin (EC 3.4.21.1), which plays a vital role in protein digestion. Chymotrypsin is a protease, specifically a serine protease, and cleaves peptide bonds formed by Phe, Leu, Trp, and Tyr in the small intestine [31]. Chymotrypsin is produced in the pancreas and is responsible for the breakdown of polypeptides in the small intestine.

Chymotrypsin has been one of the most extensively studied proteases in structural enzymology with over 16,000 PDB entries, and its crystallization was part of the 1946 Nobel Prize in Chemistry, awarded for the purification and crystallization of enzymes, including chymotrypsin [32]. Through crystallization and X-ray diffraction methods, chymotrypsin is characterized by a globular beta-protein structure primarily comprising beta-sheets forming the core beta-barrel structure (PDB ID: 4CHA). Its active site is composed of a catalytic triad consisting of Asp 102, His 57, and Ser 195, which enables the cleavage of peptide bonds [31].

### 2.4. Lyases

Lyases are a class of enzymes that cleave bonds by mechanisms other than hydrolysis or oxidation or catalyze the reverse addition of groups across double bonds. They are categorized as EC 4 in the enzyme classification system. By enabling carbon-skeleton editing and bond formation, lyases generate key structural motifs in primary and specialized metabolism, contributing to processes such as CO_2_ release and ammonia assimilation, and are widely used in synthetic biology and industry [33].

**Figure 8.**
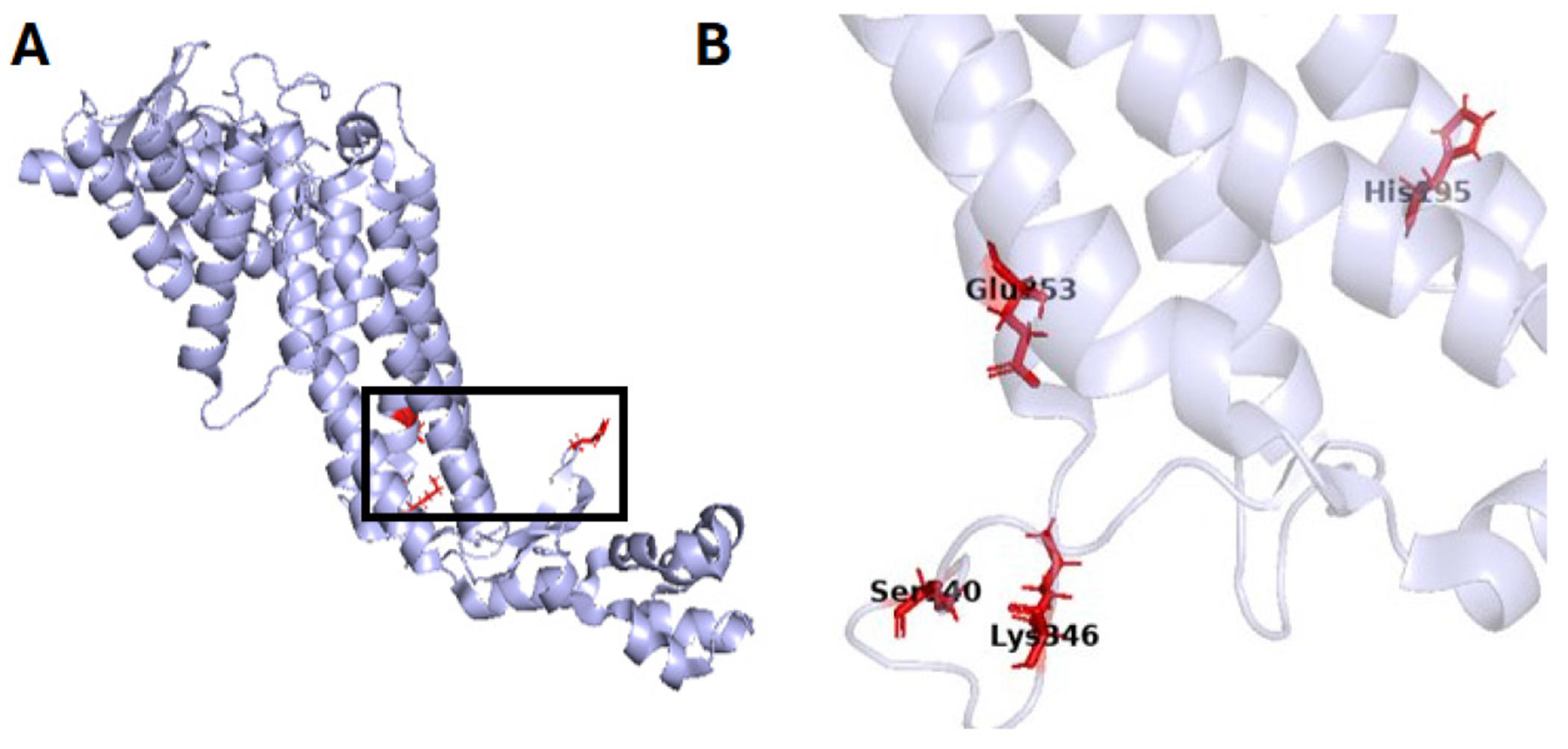
PDB structures of 6U4O (fumarase hydratase). (**A**) The protein monomer is shown in light blue. Key active-site residues are shown in red (His195, Ser340, Lys346, and Glu353). (**B**) A zoomed-in view of the active site (the region included in the black frame in panel (**A**)). His195 and Glu353 participate in the proton transfer steps of the reversible hydration–dehydration reaction. Ser340 and Lys346 orient the substrate and stabilize the transition state. All molecular graphics were generated using PyMOL 3.1.6.

One of the important enzymes in this class is fumarase (PDB ID: 6U4O) (Figure 8), also known as fumarate hydratase (EC 4.2.1.2). It is a key enzyme in the citric acid cycle that catalyzes the reversible hydration of fumarate to malate. As a member of the lyase class, specifically the hydro-lyases, fumarase plays a crucial role in cellular energy metabolism by enabling the generation of reducing equivalents (NADH, FADH_2_) that subsequently feed into the mitochondrial electron transport chain. Although most active within the mitochondrial matrix, fumarase also localizes to the cytosol, where it has been implicated in additional non-metabolic functions, including DNA repair and the cellular response to genotoxic stress [34]. This dual localization reflects the enzyme’s multifunctional significance in both metabolism and cellular homeostasis.

Importantly, mutations in the fumarate hydratase (FH) gene have been linked to hereditary leiomyomatosis and renal cell cancer (HLRCC), a rare but aggressive form of cancer. These pathogenic mutations result in enzymatic inactivation, leading to the accumulation of fumarate, which acts as an oncometabolite. Elevated fumarate levels can inhibit α-ketoglutarate-dependent dioxygenases, resulting in widespread epigenetic dysregulation, including DNA and histone hypermethylation [35]. Furthermore, fumarate-induced stabilization of hypoxia-inducible factors (HIFs) promotes a pseudohypoxic state that enhances tumorigenic potential [36,37]. Thus, fumarase has emerged as a tumor suppressor beyond its classical metabolic function, linking metabolic dysregulation directly to cancer biology [35].

The structural basis of fumarase function has been advanced by X-ray crystal structures of class II fumarase from *Thermus thermophilus* HB8 (PDB ID: 1VDK) [38]. This structure reveals a homotetrameric enzyme with a highly conserved active site, offering insights into the spatial arrangement of residues critical for substrate recognition and catalysis. The symmetrical quaternary structure facilitates substrate channeling, thereby enhancing both catalytic efficiency and regulation [39]. Crystallography has enabled researchers to elucidate the mechanisms of allosteric regulation and identify potential binding sites for small molecules. This makes fumarase an attractive target for therapeutic intervention in cancers associated with FH mutations [37]. Without crystallographic data, such detailed mechanistic insights and structure-based drug discovery initiatives would be severely limited.

### 2.5. Isomerases

Isomerases are a class of enzymes that catalyze the intramolecular rearrangement of substrates, converting a molecule into one of its isomers without changing the overall chemical formula. They are categorized as EC 5 in the enzyme classification system. By interconverting structural, geometric, or stereochemical isomers, isomerases play critical roles in carbohydrate metabolism [40].

Five families of the pseudouridine synthases (EC 5.4.99) catalyze site-specific isomerization in tRNAs and rRNA in bacteria. Pseudouridine is formed by rotation of the uridine molecule 180 degrees along the N3—C6 axis [41]. Because the C–C bond is a single sigma bond, this allows the molecule to exhibit rotational freedom and conformational flexibility [42]. In the rRNA and tRNA, the pseudouridine helps maintain the functions of mRNA decoding, ribosome assembly, processing, and translation, and also stabilizes the regional structure. Pseudouridine in the snRNA facilitates the splicing regulation through the enhancement of the spliceosomal RNA-pre-mRNA interaction [43].

**Figure 9.**
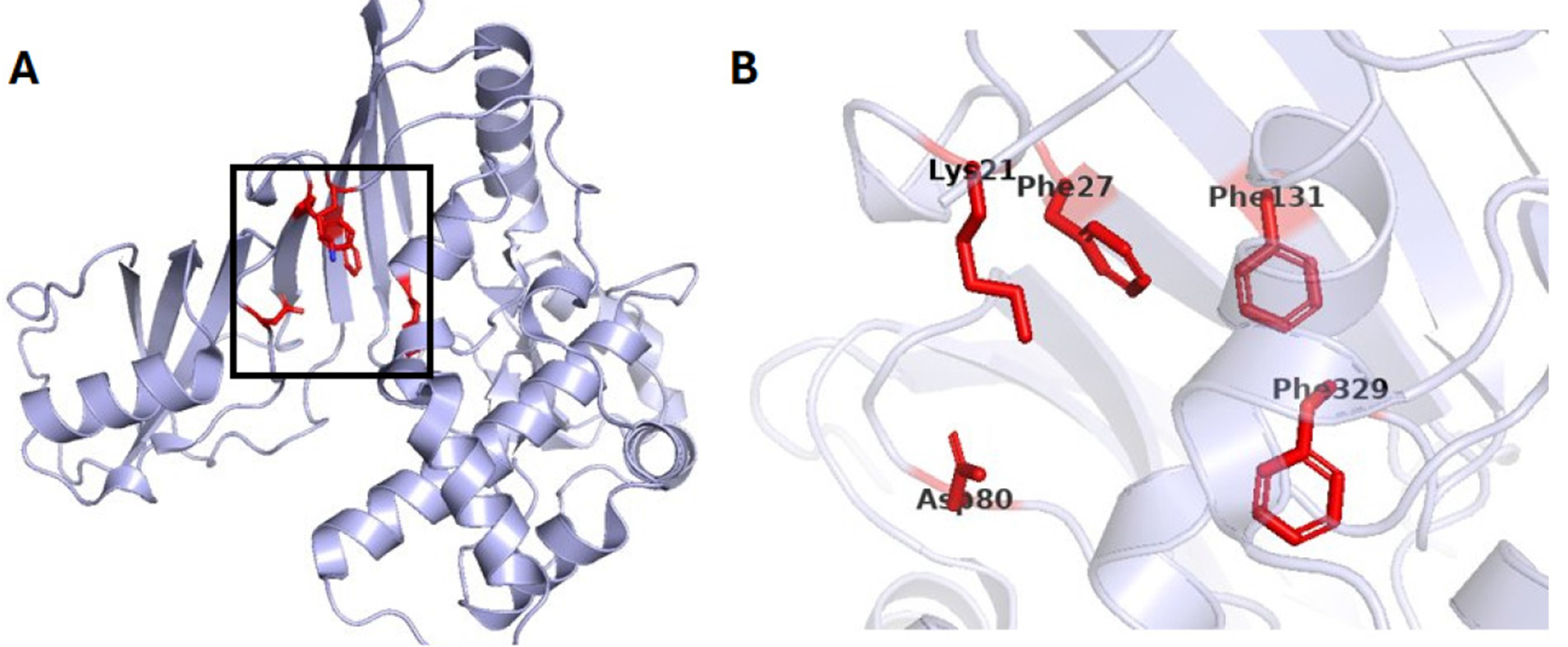
PDB structures of 1SB7 (pseudouridine synthase TruD). (**A**) The protein monomer is shown in light blue. Key active-site residues are shown in red (Lys21, Phe27, Asp80, Phe131, and Phe329). (**B**) A zoomed-in view of the active site (the region included in the black frame in panel (**A**)). Lys21 and Asp80 form the catalytic dyad isomerizing uridine to pseudouridine. The surrounding aromatic residues (Phe27, Phe131, and Phe329) stabilize the substrate base and maintain stacking interactions that facilitate C–C bond rotation during isomerization. All molecular graphics were generated using PyMOL 3.1.6.

Pseudouridine synthases can be grouped into five families, based on sequence comparison: RluA, TruA, TruB, and TruD (PDB ID: 1SB7) (Figure 9). All of the families, excluding TruA, share short chains of high sequence similarity. The three-dimensional structures of the RNA pseudouridine synthases MtTruB, EcTruB, and SnTruB have been determined by crystallography [44,45]. TruB is a cytosine synthase that controls the universally conserved Uridine 55 (U55) in the thymidine-pseudouridine-cytidine (TΨC) loop of elongator tRNAs [46], and it also serves to help the correct folding or assembly of the substrate RNAs [47].

Pseudouridine plays a vital role in the mechanisms of mRNA vaccines. When synthetic messenger RNA incorporates pseudouridine instead of the standard uridine, the resulting modified RNA molecule triggers a diminished activation of Toll-like receptors. These receptors are components of the human immune defense system that normally detect foreign RNA molecules and initiate an immune reaction. By substituting uridine with pseudouridine, the synthetic mRNA is less likely to be recognized as a threat, thereby reducing the immune system’s defensive response. A notable example of this is the use of N1-methylpseudouridine, which is present in the mRNA vaccine for the severe acute respiratory syndrome coronavirus 2 (SARS-CoV-2). It is employed instead of regular pseudouridine due to its reduced innate immune response and improved translation capacity [48].

### 2.6. Ligases

Ligases are classified as EC class 6. One important enzyme in this class is the Ubiquitin ligase (EC 6.3.2.19). These enzymes catalyze the covalent attachment of ubiquitin, a 76-amino-acid regulatory protein, to specific lysine residues on substrate proteins. This post-translational modification serves as a degradation signal, targeting tagged proteins to the 26S proteasome for controlled proteolysis. Ubiquitination occurs through a hierarchical enzymatic cascade involving E1 (ubiquitin-activating) enzymes, E2 (ubiquitin-conjugating) enzymes, and E3 (ubiquitin ligase) enzymes. Among these, E3 ligases provide substrate specificity, acting as molecular gatekeepers that determine which proteins are marked for degradation [49].

E3 ubiquitin ligases are indispensable in regulating numerous cellular processes, including cell cycle progression, signal transduction, DNA damage repair, and apoptosis. Their ability to target key regulatory proteins for degradation ensures tight control over processes that, when dysregulated, can lead to pathological states. Mutations or altered expression of E3 ligases have been implicated in cancer, autoimmune conditions, and neurodegenerative diseases like Alzheimer ’s and Parkinson’s [50]. The profound importance of this system in cell biology was recognized with the 2004 Nobel Prize in Chemistry, awarded to Aaron Ciechanover, Avram Hershko, and Irwin Rose for uncovering the mechanisms of ubiquitin-mediated protein degradation. The dynamic and reversible nature of this system also presents opportunities for targeted therapeutics in diseases involving proteostasis imbalances.

**Figure 10.**
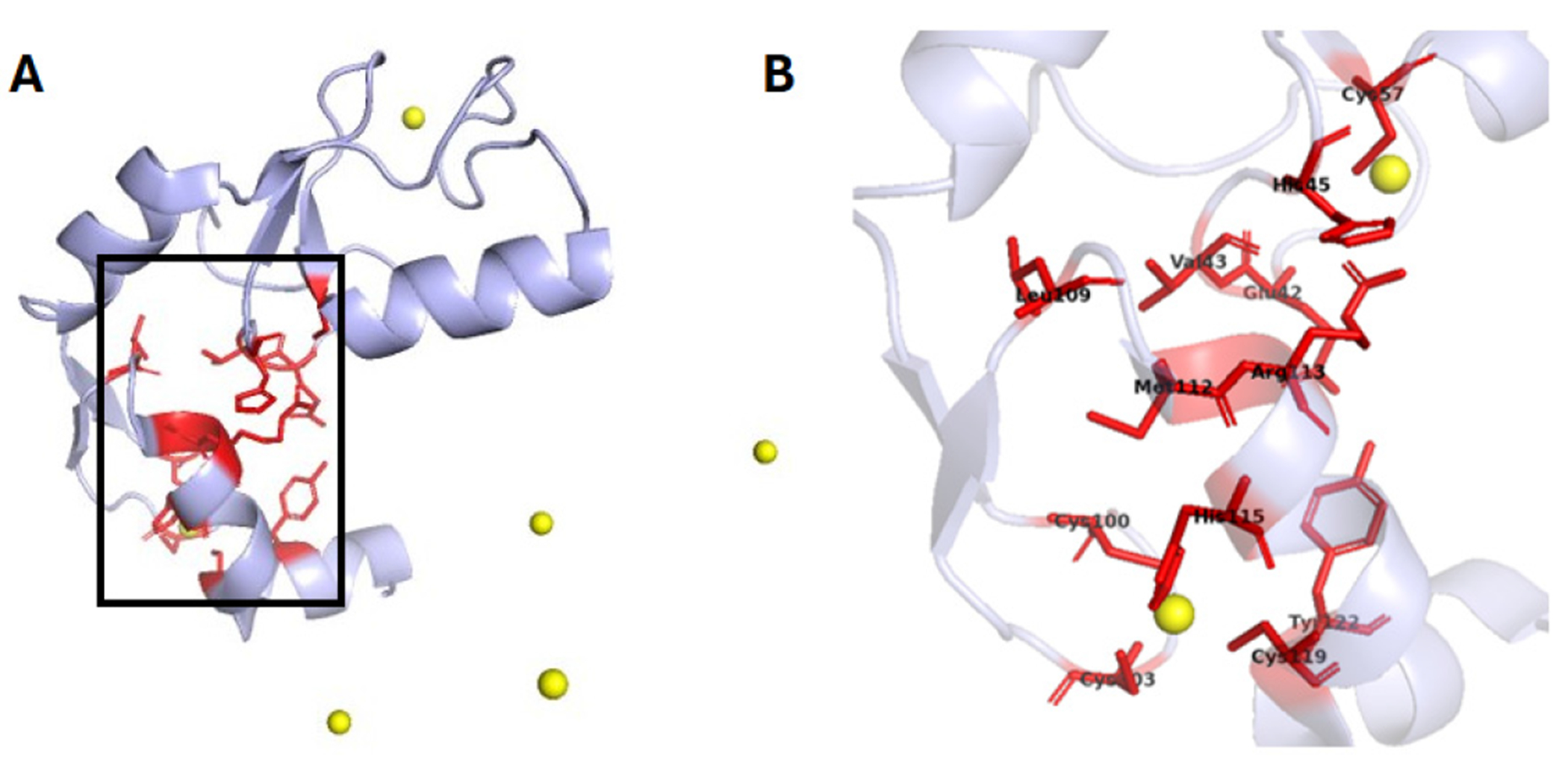
PDB structures of 5DKA (RNF125 RING + C2HC zinc finger domain). (**A**) The protein monomer is shown in light blue. Key residues are shown in red (Cys100, Cys103, His115, and Cys119 from the C2HC ZnF; Glu42, Val43, His45, and Cys57 from the RING domain; and Leu109, Met112, Arg113, and Tyr122 at the interdomain interface). Zinc ions are shown as yellow spheres. (**B**) A zoomed-in view of the RING–ZnF interface (the region included in the black frame in panel (**A**)) reveals how the coordinated Zn^2+^ ions stabilize the local fold. The RING residues and ZnF residues form the catalytic core that facilitates ubiquitin transfer to substrate lysine. All molecular graphics were generated using PyMOL 3.1.6.

Insights into the structure and function of E3 ligases have been significantly advanced through X-ray crystallography, which provides atomic-level resolution of enzyme-substrate interactions. A notable structure depicts a RING-type E3 ligase in complex with its cognate E2-conjugating enzyme and a ubiquitin molecule (PDB ID: 5DKA) (Figure 10). This structure reveals the spatial organization critical for ubiquitin transfer and substrate orientation [51,52]. Structural studies like this clarify the mechanistic basis of ligase activity and enable the rational design of small-molecule inhibitors or modulators that can selectively interfere with aberrant ubiquitination. Recent drug discovery efforts targeting E3 ligases, such as MDM2 and cereblon, rely heavily on structural information to develop proteolysis-targeting chimeras (PROTACs) and other therapeutics [53]. Crystallography remains an indispensable tool for decoding the molecular choreography of ubiquitin ligases and advancing clinical interventions.

### 2.7. Translocases

Translocases are a class of enzymes that catalyze the movement of ions or molecules across membranes, or their separation within membranes, often coupled to the hydrolysis of ATP or other energy sources. Translocases are classified as EC class 7. ATP synthase (EC 7.1.2.2) is a translocase enzyme complex located in the inner mitochondrial membrane and in the membranes of chloroplasts and bacteria. It plays a central role in oxidative phosphorylation, coupling the movement of protons (H^+^) across a membrane to the synthesis of adenosine triphosphate (ATP) from adenosine diphosphate (ADP) and inorganic phosphate (Pi). This mechanism allows cells to convert electrochemical energy generated by the proton gradient across the membrane into the chemical energy stored in ATP—the cell’s universal energy currency [54]. ATP synthase comprises two main domains: F_0_, which forms the proton channel embedded in the membrane, and F_1_, which catalyzes the formation of ATP on the matrix side [55].

The biological significance of ATP synthase is immense. Without it, aerobic organisms would be unable to harness metabolic fuel efficiently. The enzyme is considered one of biology’s most evolutionarily conserved and functionally essential molecular machines. The significance of its function was recognized by the 1997 Nobel Prize in Chemistry, awarded to Paul D. Boyer and John E. Walker for elucidating its rotary catalytic mechanism [56]. Their research demonstrated that the energy from proton flow is not used directly to form ATP, but rather to induce conformational changes in the enzyme that drives ATP synthesis, a concept known as the binding change mechanism. The enzyme’s remarkable rotary mechanism was found to operate with high efficiency, converting the proton motive force into chemical energy, which is essential for life in all aerobic organisms [56]. This research has paved the way for novel therapies targeting mitochondrial dysfunction and shed light on fundamental processes such as cellular aging and metabolic disease.

**Figure 11.**
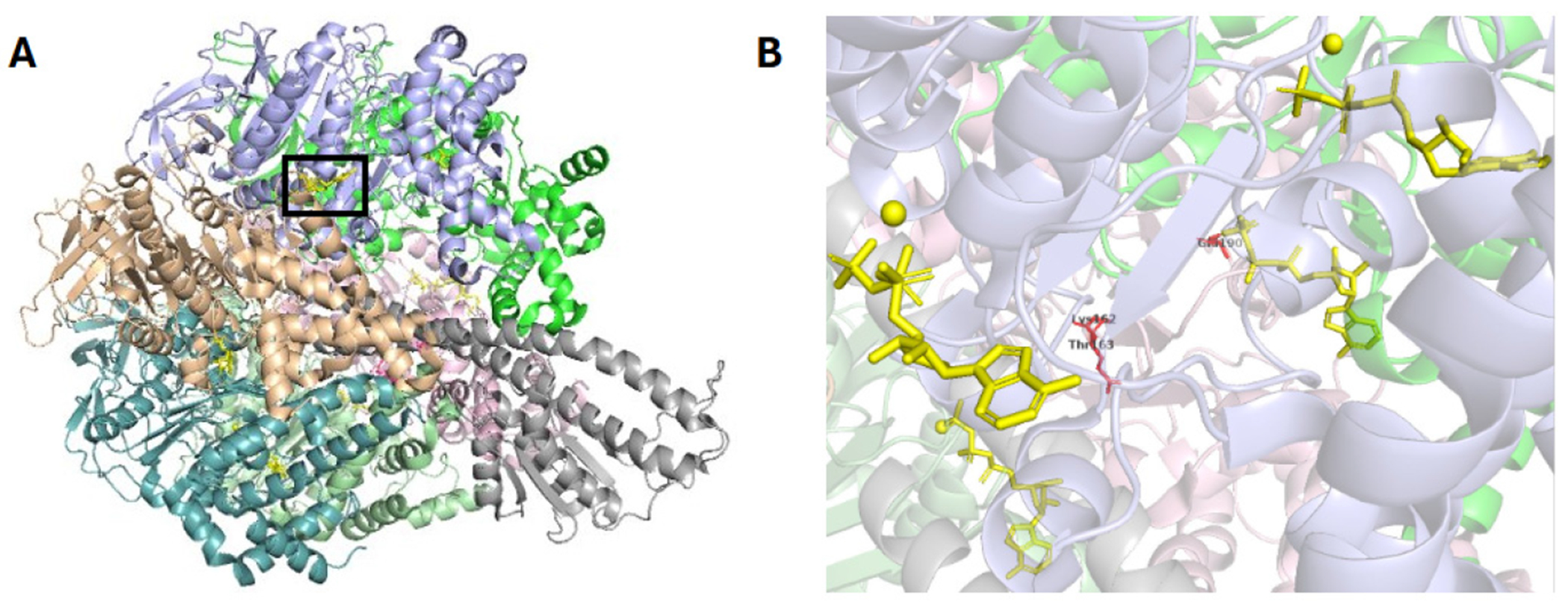
PDB structures of 7L1Q (F_1_-ATPase catalytic domain). (**A**) Seven subunits of the α_3_β_3_γ subcomplex are each shown in distinct colors. Bound nucleotides and Mg^2+^ ions are shown in yellow. (**B**) A zoomed-in view of one β-subunit active site (β-chain) (the region included in the black frame in panel (**A**)). Key catalytic residues are colored red (Thr163, Glu190). These residues participate in ATP hydrolysis. Conformational changes in the β-subunit drive the 120° rotation of the central γ-subunit characteristic of the F_1_-ATPase rotary mechanism. All molecular graphics were generated using PyMOL 3.1.6.

The understanding of ATP synthase’s function was resolved through cryo-electron microscopy (Cryo-EM), which enabled visualization of its molecular structure at atomic resolution. Structural insights from crystallized enzymes (PDB ID: 7L1Q) (Figure 11) revealed the precise arrangement of subunits and how rotation within the F0-F1 complex contributes to ATP synthesis [57,58]. By freezing the enzyme in specific states, researchers could map its dynamic conformational changes, which are crucial for understanding how it harnesses proton motive force to catalyze ATP formation. Without structural biology tools like X-ray crystallography, this depth of mechanistic insight would not have been possible. The advances in Cryo-EM have also made large and dynamic complexes accessible, and these complex studies are contributing more and more to our understanding of the mechanisms in the future.

## 3. Discussion

The diverse functions of enzymes can often be explained by first uncovering the details of their three-dimensional structures. For various EC groups, obtaining high-resolution structural data has been crucial in understanding catalytic reactions, including substrate specificity and necessary orientations. Such insights serve as steppingstones to drug design, treatment methods, and understanding the molecular machinery of life.

In modern structural enzymology, three principal techniques are X-ray crystallography, nuclear magnetic resonance (NMR) spectroscopy, and cryo-electron microscopy (Cryo-EM). Besides these experimental methods, modern computational tools (AlphaFold, RosettaFold, ESMFold, and Boltz-2, etc.) also complement them in predicting structures, dynamics, and further help in modeling and macromolecule design. In the sections that follow, we outline what each technique uniquely resolves and how we integrate structure-prediction tools with these data, then apply this framework to our EC-class case studies.

X-ray crystallography remains the most widely used for determining high-resolution atomic structure. It provides unparalleled atomic detail and furthers the field of structural enzymology. This technique enables us to visualize active sites and conformational changes following ligand binding, including the elucidation of the induced fit model in action, which maps out enzyme-substrate and enzyme-inhibitor interactions. Despite its immense power, however, crystallography faces inherent challenges, particularly when attempting to capture the dynamic and transient nature of enzymatic reactions. The minute and specific mechanistic features of enzymes often come with unique hurdles. For instance, enzymatic reactions proceed through a series of rapid, ephemeral, intermediate states, and transition states. Traditional X-ray crystallography, by its nature, captures a time-averaged view that is static. In contrast, the timescales of catalytic events are significantly faster than the seconds to minutes typically needed for X-ray data recording and collection. In recent years, time-resolved crystallography has also provided insights into such transient processes, revealing intermediates in catalytic cycles [59,60].

Nevertheless, crystallography remains indispensable for structure-based drug design, provided that the target protein can be crystallized at good resolution. The Protein Data Bank (PDB) contains many examples of one enzyme with multiple ligand-bound structures that have been solved at high resolution, offering atomic-level insights. Furthermore, proteins too small for cryo-electron microscopy (Cryo-EM) often rely on crystallographic studies to obtain detailed structural information.

Nuclear magnetic resonance (NMR) spectroscopy offers a powerful alternative for elucidating enzyme structures and dynamics in solution. Unlike crystallography, which requires ordered crystals, NMR enables structural studies under near-physiological conditions, allowing for the capture of transient states and conformational changes over time. Although limited to proteins generally under 50 kDa due to signal complexity and sensitivity issues, NMR remains indispensable for probing flexible regions, identifying dynamic allosteric sites, and validating binding interactions of small-molecule ligands in enzymes.

The technique of cryo-electron microscopy (Cryo-EM) has begun to overcome these limitations of crystallography, especially in capturing large, flexible protein complexes. It provides structural insights into previously intractable dynamic systems, such as enzyme-substrate intermediates. Unlike X-ray crystallography, Cryo-EM images vitrify particles directly in thin ice, eliminating the need for crystals. Single particle analysis can sort heterogeneous conformations from the same dataset, enabling reconstructions of multiple functional states along a reaction pathway. Compared with NMR, Cryo-EM is not fundamentally limited by size and is now gradually reaching higher resolutions. Moreover, cryo-electron tomography (Cryo-ET) extends these advantages in situ, revealing the organization of enzymes within intact cells and membranes, contexts that purified crystals or solution NMR cannot capture.

Together, these experimental methods reveal atomic details and conformational changes, but they rarely capture the full dynamics or all relevant complexes in vivo. Modern computational approaches bridge this gap: physics-based modeling and machine learning integrate the restraints to build ensemble models and guide mutagenesis/ligand design. These computationally based predictions improved mechanistic insight and enzyme engineering.

In 2021, AlphaFold was applied to predict structures for essentially the entire human proteome and hundreds of millions of proteins from other organisms, including many proteins (such as human membrane enzymes) that have no prior solved structures [61]. One of AF2’s important predictions is human glucose-6-phosphatase (G6Pase-α), a membrane-bound enzyme that catalyzes the final step of glucose synthesis. No crystal or EM structure of this enzyme existed, yet AlphaFold confidently predicted a nine-transmembrane-helix fold with the catalytic site facing the lumen via a solvent-accessible tunnel. In the CASP14 blind challenge, AlphaFold2 achieved a median backbone RMSD error of around 1 Å [62]. Following DeepMind’s breakthrough, other AI tools have emerged. The more recent RoseTTAFold All-Atom (RFAA) framework extends the original RoseTTAFold architecture to model proteins, nucleic acids, ligands, and their complexes at full-atom resolution within a single, unified deep learning framework [63]. RFAA jointly reasons over all atoms during training and inference, enabling direct prediction of chemically detailed structures for diverse biomolecular assemblies. This capability enables the modeling of enzyme–ligand and protein–nucleic acid complexes at an atomic level, supporting downstream applications in ligand docking, binding-site characterization, and the design of active-site mutations. ESMFold, developed by Meta AI, utilizes a large protein language model to predict structures directly from single sequences, eliminating the need for multiple sequence alignments. This enables ultra-fast predictions for millions of proteins, albeit at a slightly reduced accuracy compared to AF2 [64]. Together, these AI-based predictors provide a structural foundation that also increasingly guides enzyme engineering efforts, from rational active-site redesign to de novo enzyme creation.

With high-quality 3D predictions in hand, scientists can engineer enzymes by identifying key residues and guiding directed evolution. AlphaFold2 was used to model a PET-degrading enzyme and revealed how certain mutations would increase the flexibility and size of its substrate-binding groove [65]. Incorporating those mutations produced a “TurboPETase” variant capable of near-complete plastic depolymerization, which outperformed earlier enzymes [65]. In synthetic biology, structure predictions of enzymes in metabolic pathways enable the refinement of active sites and interfaces to enhance pathway flux or alter substrate specificity, often in conjunction with ancestral sequence information to maintain protein foldability.

Boltz-2 was released in 2025 by researchers at MIT’s Computer Science and Artificial Intelligence Laboratory in collaboration with Recursion. Unlike previous models that only predicted 3D structures, Boltz-2 incorporates an affinity module that can predict how strongly small molecules bind to their protein targets, which was a critical bottleneck in drug discovery. Its performance is comparable to that of expensive free energy perturbation (FEP) simulations, while being over 1000 times faster [66]. This breakthrough enables large-scale virtual screening and affinity-guided molecular design workflows that were previously computationally prohibitive, allowing researchers to rapidly screen millions of compounds and guide AI models to design optimized drug candidates [66].

By combining AI-driven structural prediction with experimental techniques and evolutionary insights, researchers can now design enzymes with unprecedented precision, accelerating innovations from sustainable biocatalysts to therapeutic developments and enhancing the impact of structure-guided enzymology.

Looking toward the future, the convergence of experimental and computational structural biology promises to revolutionize our approach to understanding enzyme mechanisms, facilitating therapeutic discovery, and enabling precision medicine. The integration of high-resolution crystallographic data with AI-driven predictions and dynamic simulations creates a framework where experimental structures serve as a ground truth for validating computational models, while AI tools guide experimental design by predicting which variants, conformations, or binding partners merit investigation. This cooperative relationship is exemplified by emerging approaches that combine traditional X-ray crystallography with machine learning algorithms to automatically identify cryptic binding pockets, predict the effects of missense variants on enzyme stability and function, and design novel enzyme variants with enhanced therapeutic properties. The trajectory from ancient crystallization practices to modern AI-powered enzyme engineering represents not merely technological advancement but a fundamental transformation in how we approach the molecular basis of life and disease, promising a future where the atomic-level insights first revealed by scientists can be systematically improved to engineer solutions for human health challenges.

## Supplementary Material

Supplementary Table

The following supporting information can be downloaded at: https://www.mdpi.com/article/10.3390/cryst15110966/s1, Table S1: Overview of all enzymes and their number of publications. Data from the Research Collaboratory for Structural Bioinformatics (RCSB) Protein Data Bank (PDB), updated by 31 July 2025; Table S2: Overview of the Oxidoreductase enzyme class, their function, and total PDB structures. Data from RCSB PDB, updated by 31 July 2025; Table S3: Overview of the Transferases enzyme class, their function, and total PDB structures. Data from RCSB PDB, updated by 31 July 2025; Table S4: Overview of the Hydrolases enzyme class, their function, and total PDB structures. Data from RCSB PDB, updated by 31 July 2025; Table S5: Overview of the Lyases enzyme class, their function, and total PDB structures. Data from RCSB PDB, updated by 31 July 2025; Table S6: Overview of the Isomerases enzyme class, their function, and total PDB structures. Data from RCSB PDB, updated by 31 July 2025; Table S7: Overview of the Ligases enzyme class, their function, and total PDB structures. Data from RCSB PDB, updated by 31 July 2025; Table S8: Overview of the Translocases enzyme class, their function, and total PDB structures. Data from RCSB PDB, updated by 31 July 2025. For the generation of Tables S1–S8, data were obtained from the RCSB Protein Data Bank using a custom Python 3.0 script. The script queried EC numbers to extract counts of unique enzyme commission classes and total associated PDB entries. The Python script is available on GitHub: “https://github.com/Liang-Research-Group/Automation” (accessed on 1 October 2025). See the README file for specific instructions. Representative coordinates were fetched from the RCSB Protein Data Bank directly in PyMOL using the fetch command, and protein figures were prepared in PyMOL 3.1.6 (The PyMOL Molecular Graphics System, Schrödinger, LLC).

## Abbreviations

The following abbreviations are used in this manuscript:

PDB: Protein Data Bank
NAD^+^: nicotinamide adenine dinucleotide (oxidized form
NADH: nicotinamide adenine dinucleotide with hydrogen (reduced form)
OYE: old yellow enzyme
M.W.: molecular weight
PEP: phosphoenolpyruvate
PTS: phosphotransferase system
HPr: histidine phosphocarrier protein
RNase III: Ribonuclease 3
FH: fumarate hydratase
HLRCC: hereditary leiomyomatosis and renal cell cancer
HIFs: hypoxia-inducible factors
U55: Uridine 55
TΨC: thymidine-pseudouridine-cytidine loop
snRNA: small nuclear RNA
SARS-CoV-2: severe acute respiratory syndrome coronavirus 2
E1: ubiquitin-activating enzyme
E2: ubiquitin-conjugating enzyme
E3: ubiquitin ligase enzyme
PROTACs: proteolysis-targeting chimeras
ATP: adenosine triphosphate
ADP: adenosine diphosphate
Pi: inorganic phosphate
NMR: nuclear magnetic resonance
Cryo-EM: cryo-electron microscopy
Cryo-ET: cryo-electron tomography
AF2: AlphaFold2
RMSD: root-mean-square deviation
RFAA: RoseTTAFold All-Atom
PET: polyethylene terephthalate
FEP: free energy perturbation
RCSB: Research Collaboratory for Structural Bioinformatics
HEW: hen egg-white (lysozyme
