# Supplementary material for: The Crystallography of Enzymes: A Retrospective and Beyond
Source: Crystals (Basel). Author manuscript; Available in PMC 2026 Jul 8. (PMC13341174; doi:10.3390/cryst15110966)
Supplement: Supplementary Table [file NIHMS2187263-supplement-Supplementary_Table.pdf]

Supplementary Tables

Table S1. Overview of all enzymes and their number of publications. Data from the Research Collaboratory for Structural Bioinformatics (RCSB) Protein Data Bank (PDB), updated by 07/31/2025.

EC number	Name	# of PDB Publications
1	Oxidoreductases	21193
2	Transferases	40773
3	Hydrolases	54489
4	Lyases	9785
5	Isomerases	5658
6	Ligases	4143
7	Translocases	2524

Table S2. Overview of the Oxidoreductase enzyme class, their function, and total PDB structures. Data from RCSB PDB, updated by 07/31/2025

EC Number	Name	Bonds they act on	# of PDB structures
1.1	Alcohol oxidoreductases	CH-OH group of donors	3786
1.2	Aldehyde/oxo oxidoreductases	aldehyde or oxo group of donors	1239
1.3	CH-CH oxidoreductases	CH-CH group of donors	1511
1.4	Amino acid oxidoreductases	CH-NH2 group of donors	643
1.5	Oxidoreductases acting on the CH-NH group of donors	CH-NH group of donors	1,296
1.6	Oxidoreductases acting on NADH or NADPH	NADH or NADPH	926
1.7	Nitrogenous donor	Nitrogenous compounds as donors	1,396
1.8	Sulfur oxidoreductases	Sulfur group of donors	870
1.9	Oxidoreductases acting on heme group of donors.	Heme group of donors	172
1.10	Oxidoreductases acting on diphenols and related substances as donors	Diphenols and related substances as donors	587
1.11	Peroxidases	Peroxide as acceptor	2026

1.12	Oxidoreductases acting on hydrogen as donor	Hydrogen as donor	243
1.13	Monooxygenases	Single donors with incorporation of molecular oxygen	937
1.14	Dioxygenases	Paired donors with incoporation of reduction of molecular oxygen	4,629
1.15	Oxidoreductases acting on superoxide as acceptor	Superoxide as acceptor	521
1.16	Oxidoreductases acting on oxidizing metal ions	Oxidizing metal ions	653
1.17	Oxidoreductases acting on CH or CH2 groups	CH or CH2 groups	552
1.18	Oxidoreductases acting on iron-sulfur proteins as donors	Iron-sulfur proteins as donors	346
1.19	Oxidoreductases acting on reduced flavodoxin	Reduced flavodoxin	11
1.20	Oxidoreductases acting on phosphorus or arsenic in donors	Phosphorus or arsenic in donors	90
1.21	Oxidoreductases catalyzing the reaction X-H + Y-H = X-Y	Catalyzing the reaction X-H + Y-H = X-Y	121
1.22	Oxidoreductases acting on halogen in donors	Halogen in donors	4
1.23	Oxidoreductases reducing C-O-C group as acceptor	Reducing C-O-C group as acceptor	13
1.97	Other oxidoreductases	Sole subclass for oxidoreductases that do not	158

		belong in the other subclasses	

Table S3. Overview of the Transferases enzyme class, their function, and total PDB structures. Data from RCSB PDB, updated by 07/31/2025.

EC Number	Name	Bonds they act on	# of PDB structures
2.1	Single carbon transferases	Single-carbon groups	4486
2.2	Aldehyde and ketone transferases	Aldehyde or ketone groups	295
2.3	Acyltransferases	Acyl groups or groups that become alkyl groups during transfer	6533
2.4	Glycosyl, hexosyl, and pentosyl transferases	Glycosyl groups, as well as hexoses and pentoses	4209
2.5	Alkyl and aryl transferases	Alkyl or aryl groups, other than methyl groups	2495
2.6	Nitrogenous transferases	Nitrogenous groups	963
2.7	Phosphorus transferases	Phosphorus-containing groups; subclasses are based on the acceptor (e.g. alcohol, carboxyl, etc.)	22017
2.8	Sulfur transferases	Sulfur-containing groups	505
2.9	Selenium transferases	Selenium-containing groups	19
2.10	Metal transferases	Molybdenum or tungsten	59

Table S4. Overview of the Hydrolases enzyme class, their function, and total PDB structures. Data from RCSB PDB, updated by 07/31/2025.

EC Number	Name	Bonds they act on	# of PDB structures
3.1	Estererases, nucleases,	Ester bonds	13385

	phosphodiesterases, lipases, phosphatases		
3.2	DNA glycosylases, glycoside hydrolase	Sugars	8954
3.3	Hydrolases acting on ether bonds	Ether bonds	414
3.4	Proteases, peptidases	Peptide bonds	17153
3.5	Hydrolases acting on carbon-nitrogen bonds other than peptide bonds.	Carbon-nitrogen (non-peptide) bonds	5312
3.6	Acid anhydrides	Acid anhydride hydrolases, including helicases and GTPase	10790
3.7	Hydrolases acting on carbon-carbon bonds	Carbon-carbon bonds	88
3.8	Hydrolases acting on halide bonds	Halide bonds	296
3.9	Hydrolases acting on phosphorus-nitrogen bonds	Phosphorus-nitrogen bonds	108
3.10	Hydrolases acting on sulfur-nitrogen bonds	Sulfur-nitrogen bonds	2
3.11	Hydrolases acting on carbon-phosphorus bonds	Carbon-phosphorus bonds	13
3.12	Hydrolases acting on sulfur-sulfur bonds	Sulfur-sulfur bonds	2
3.13	Hydrolases acting on carbon-phosphorus bonds	Carbon-phosphorus bonds	170

Table S5. Overview of the Lyases enzyme class, their function, and total PDB structures. Data from RCSB PDB, updated by 07/31/2025.

EC Number	Name	Bonds they act on	# of PDB structures
4.1	Carbon-Carbon Lyases	Carbon-Carbon	2,595
4.2	Carbon-Oxygen Lyases	Carbon-Oxygen	5245
4.3	Carbon-Nitrogen Lyases	Carbon-Nitrogen	556

4.4	Carbon-Sulfur lyases	Carbon-Sulfur	365
4.5	Carbon-Halide lyases	Carbon-Halide	14
4.6	Phosphorus-Oxygen lyase	Phosphorus-Oxygen	939
4.7	Carbon-Phosphorus lyases	Carbon-phosphorus	6
4.8	Hydro-lyases	Nitrogen-oxygen	12
4.98	Forming coordination complexes	ATP-independent chelatases	38
4.99	Other lyases	Other Lyases	127

Table S6. Overview of the Isomerases enzyme class, their function, and total PDB structures. Data from RCSB PDB, updated by 07/31/2025.

EC Number	Name	Bonds they act on	# of PDB structures
5.1	Racemases and Epimerases	Stereochemistry at chiral carbons	757
5.2	Cis-trans Isomerases	Cis-trans isomers of alkenes and cycloalkanes	1268
5.3	Intramolecular Oxidoreductases	Electron transfer within a molecule	1589
5.4	Intramolecular Transferases	Transfer of functional groups within a molecule	771
5.5	Intramolecular Lyases	Group elimination leaving a double bond or ring break	122
5.6	Macromolecular Isomerases	Conformational and topological changes in macromolecules	1141

Table S7. Overview of the Ligases enzyme class, their function, and total PDB structures. Data from RCSB PDB, updated by 07/31/2025.

EC Number	Name	Bonds they act on	# of PDB structures
6.1	Ligases forming carbon–oxygen bonds	Carbon-oxygen bonds	1217

6.2	Ligases forming carbon–sulfur bonds	Carbon-sulfur bonds	366
6.3	Ligases forming carbon–nitrogen bonds	Carbon-nitrogen bonds	2213
6.4	Ligases forming carbon–carbon bonds	Carbon-carbon bonds	196
6.5	Ligases forming phosphoric ester bonds	Phosphoric ester bonds	222
6.6	Ligases forming nitrogen–metal bonds	Nitrogen-metal bonds	16

Table S8. Overview of the Translocases enzyme class, their function, and total PDB structures. Data from RCSB PDB, updated by 07/31/2025.

EC Number	Name	Functions	# of PDB structures
7.1	Translocases catalyzing the translocation of hydrons	Catalyzing the movement of hydrons (H^+ ions) across biological membranes	1353
7.2	Translocases catalyzing the translocation of inorganic cations	Catalyzing the translocation of inorganic cations and their chelates	442
7.3	Translocases catalyzing the translocation of inorganic anions	Catalyzing the translocation of inorganic anions	10
7.4	Translocases catalyzing the translocation of amino acids and peptides	Catalyzing the translocation of amino acids and peptides	143
7.5	Translocases catalyzing the translocation of carbohydrates and derivatives	Catalyzing the translocation of carbohydrates and their derivatives	105
7.6	Translocases catalyzing the translocation of other compounds	Catalyzing the translocation of other compounds	449
